# Electrophysiology Fellowship Survival Guide

**DOI:** 10.1016/j.hrcr.2024.06.006

**Published:** 2024-07-15

**Authors:** John J. Lee

**Affiliations:** Baptist Health Lexington, Lexington, Kentucky

Congratulations to all the incoming electrophysiology (EP) fellows. The last chapter of your official medical training is finally here. Here are a few words of advice on how to make your last fellowship the best training experience.

(1) **Know where the bathroom is.** You might think I am being facetious, but this is one of the most practical tips that I can give. EP procedures are long and unpredictable. Knowing where your safe space is will get you out of trouble.
(2) **Be kind to all staff.** Your lab staff, nurses, mappers, and device representatives are your new family. You will spend more time with this group of people than you will with your own family. Get to know them and treat them with respect—and, in turn, they will take care of you.
(3) **Scrub into as many cases as you can.** Device implantation techniques and catheter maneuvers are learned skills. Like any motor skill, the more you do it, the better you will become. You have to develop your muscle memory through repetition. Start with small successes—getting good accesses, closing the pocket well, etc. Not only scrub into many different procedures, but also scrub in with different attendings. Soon, you will see each attending has his or her own approach to a same case. Learning different approaches will help you to develop your own approach.
(4) **Be curious around the EP lab.** In addition to catheter manipulations or device implantation techniques, there are other skills to be learned in the EP lab. In between your cases, roam around to other labs; you never know what you will learn. Offering to help run a stimulator will allow you an opportunity to master skills such as performing a comprehensive EP study and different maneuvers. You should also learn how to set your room up from start to finish, including how to set up a table, connect cables, apply patches appropriately, configure the recording system to your preference, etc. Take time to learn the valuable skills that are not reflected in the academic curriculum, but that are necessary for troubleshooting and workflow efficiency.
(5) **Learn how to be an EP consultant.** I will be the first to admit that when I started my fellowship training, I wanted to skip my inpatient week or clinic. I thought I paid my dues during my cardiology fellowship. But looking back, I am glad my fellowship provided these opportunities. As an EP fellow, I was able to run my own service and learned how to talk to patients, families, and other providers. Through repeated decision-making opportunities, I have learned to make decisions quickly and confidently. Moreover, I have learned to effectively communicate those decisions to families and other providers.
(6) **Prepare yourself for complications.** Unfortunately, in our profession, complications are inevitable. Fortunately, as a trainee, you are in an environment where degrees of mistakes are allowed. Learn from your mistakes. Learn how to avoid mistakes. Learn to fix your mistakes. More importantly, learn how to talk to your patients or their family about complications; or even just observe your attending have that conversation. After training, when patients become YOUR patients, you take on the full responsibility of them—from the moment they enter the hospital to the moment they get discharged. Conversations after adverse events will always be difficult, but having experience will help you navigate through these events more easily.
(7) **Read, read, and read.** Electrophysiology is a separate language from cardiology. There are numerous topics and concepts that you will have to master from the start. My mentor, Dr Okabe, gave me this list to start from: https://www.escardio.org/static-file/Escardio/Education-Subspecialty/Certification/EHRA/Invasive%20electrophysiology/EHRA_EP_ReadingList.pdf.
(8) **Take care of yourself.** You will only be as good a doctor as your health will allow you to be. I had a major health scare during my training and since then I have been drawing back on that moment to appreciate the second opportunity. Take care of yourself so that you can take better care of your patients. Also, do not forget to take care of and appreciate your loved ones.

Again, congratulations, and I hope you take full advantage of these next 2 years!

## Disclosures

No conflicts of interest to disclose at this time.

